# Enhanced annealing of mismatched oligonucleotides using a novel melting curve assay allows efficient *in vitro *discrimination and restriction of a single nucleotide polymorphism

**DOI:** 10.1186/1472-6750-11-83

**Published:** 2011-08-30

**Authors:** Stephen R Doyle, Chee Kai Chan, Warwick N Grant

**Affiliations:** 1 La Trobe Institute for Molecular Science, La Trobe University, Bundoora, Australia

## Abstract

**Background:**

Many SNP discrimination strategies employ natural restriction endonucleases to discriminate between allelic states. However, SNPs are often not associated with a restriction site and therefore, a number of attempts have been made to generate sequence-adaptable restriction endonucleases. In this study, a simple, sequence-adaptable SNP discrimination mechanism between a 'wild-type' and 'mutant' template is demonstrated. This model differs from other artificial restriction endonuclease models as *cis- *rather than *trans-*orientated regions of single stranded DNA were generated and cleaved, and therefore, overcomes potential issues of either inefficient or non-specific binding when only a single variant is targeted.

**Results:**

A series of mismatch 'bubbles' that spanned 0-5-bp surrounding a point mutation was generated and analysed for sensitivity to S1 nuclease. In this model, generation of oligonucleotide-mediated ssDNA mismatch 'bubbles' in the presence of S1 nuclease resulted in the selective degradation of the mutant template while maintaining wild-type template integrity. Increasing the size of the mismatch increased the rate of mutant sequence degradation, until a threshold above which discrimination was lost and the wild-type sequence was degraded. This level of fine discrimination was possible due to the development of a novel high-resolution melting curve assay to empirically determine changes in Tm (~5.0°C per base-pair mismatch) and to optimise annealing conditions (~18.38°C below Tm) of the mismatched oligonucleotide sets.

**Conclusions:**

The *in vitro *'cleavage bubble' model presented is sequence-adaptable as determined by the binding oligonucleotide, and hence, has the potential to be tailored to discriminate between any two or more SNPs. Furthermore, the demonstrated fluorometric assay has broad application potential, offering a rapid, sensitive and high-throughput means to determine Tm and annealing rates as an alternative to conventional hybridisation detection strategies.

## Background

Single nucleotide polymorphisms (SNPs) play a significant role in the genetic basis for many acquired and inherited genetic disorders. This has driven the development of protocols to characterise SNPs, to both detect novel polymorphisms and discriminate between allelic states, including SNPs implicated in disease. A number of these approaches employ restriction endonucleases to discriminate between sequences. Although naturally occurring restriction endonucleases are highly efficient at cleaving dsDNA at specific locations and have been used successfully to discriminate polymorphisms such as restriction fragment length polymorphisms (RFLPs), many SNPs are not associated with a natural restriction site. For other applications such as cloning, restriction endonucleases are used to great effect, however, a restriction site may not be conveniently located, or, too many restriction sites may exist within a defined sequence. To overcome these limitations, a number of efforts have been made to develop endonucleases that do not recognise a single specific DNA motif, but are sequence-adaptable.

The first generation of these sequence-adaptable endonucleases involved the use of the DNA strand-invading peptide nucleic acids (PNAs) with the ssDNA-specific S1 nuclease [1], a combination which has since been adapted in colorimetric [2-4] and fluorometric [5] assays for SNPs detection and cloning [6], including the isolation of large DNA fragments from entire *E. coli *genomic templates [7]. The cleavage mechanism typically employs two PNAs to mediate the induction of sequence-specific stretches of ssDNA in a dsDNA template that can form a substrate for a ssDNA-specific endonuclease. The orientation of the two strand-invading PNAs relative to each other determines the location and size of the ssDNA region, with the majority of reports employing two *trans*-orientated PNAs that partially overlap and therefore form single-stranded regions on both strands of DNA. This strategy is suitable for a range of restriction-based applications where complete cleavage of the sample is desired. However, when this approach is applied as a means to discriminate between two sequences that differ by a SNP, the ability to distinguish relies solely on the PNA binding to one sequence variant but not the other (i.e., the PNA binds to the mutant sequence but not the wild-type, due to instability of PNA binding in the presence of a base mismatch), rather than specific recognition of the SNP itself. This approach therefore becomes problematic when non-specific binding occurs either at the site of interest in the non-targeted sequence or at any other nonspecific site, as the binding event produces nuclease susceptible ssDNA regions regardless of the sequence that it binds to. Consequently, to improve the specificity of this approach, the discrimination mechanism needs to rely not on the presence or absence of the DNA binding molecule, but rather the presence or absence of the SNP itself.

In this investigation, the development of a novel adaptation of an S1 nuclease-mediated cleavage mechanism is described whereby DNA cleavage is not mediated by the presence or absence of the binding molecule, but by the induction of small ssDNA 'bubbles' surrounding a SNP. In this model, inducible mismatches surrounding the mutation site (created by a semi-complementary oligonucleotide in *cis- *orientation) generated a mismatch 'bubble' that was exaggerated in the mutant sequence relative to the wild-type sequence, which thereby increased the susceptibility of the mutant sequence to DNA strand cleavage by the ssDNA-specific S1 nuclease. Increasing the 'bubble' size resulted in an increase in the cleavage of the mutant sequence relative to the wild-type. Demonstration of this mechanism was made possible by the development of a novel melting curve assay to empirically calculate Tm and annealing rates of complimentary and mismatched oligonucleotides. This *in vitro *'cleavage bubble' model can be easily modified to target any site by changing the sequence of the binding oligonucleotide and hence, has the potential to be tailored to discriminate between any two or more SNPs.

## Results and Discussion

This investigation presents a new *in vitro *SNP discrimination mechanism, demonstrated by discriminating between the wild-type and mutant sequence of the mitochondrial A8344G MERRF mutation. This mutation was chosen as a target as it is one of the most common pathological mtDNA heteroplasmic variants affecting mt-tRNA^Lys ^synthesis [8] and that PNA sequence discrimination of this SNP has been previously demonstrated [9-11]. The discrimination mechanism presented was based on S1 nuclease-mediated cleavage of single stranded 'bubbles' created by induced mismatches between an annealed oligonucleotide and target mtDNA sequence. However, this mechanism differs from previous S1 nuclease/PNA cleavage models, as *cis- *rather than *trans-*orientated regions of single stranded DNA were generated and cleaved (See Additional file 1) and that both wild-type and mutant templates are targeted by the bubble-forming oligonucleotides, rather than exclusively targeting one sequence and not the other, a common approach in many PNA-based SNP discrimination strategies. Therefore, it is the bubble size difference between the mutant and wild-type sequence, rather that the presence or absence of the binding molecule, that dictates cleavage.

### Design and generation of nuclease-sensitive 'bubbles'

To assess the efficacy of S1 nuclease-mediated sequence discrimination between the wild-type and mutant templates, a series of gradually increasing mismatches were generated to determine both the minimal 'bubble' size that would confer nuclease sensitivity on the mutant template while leaving the wild-type template uncleaved, and, the maximum size of the mismatch 'bubble' that would render both templates sensitive to the nuclease treatment. Furthermore, since any mismatch configurations that existed within the minimum and maximum defined 'bubbles' would create different sized regions of ssDNA, this analysis would provide a means to observe variation in the cleavage rate due to differential substrate recognition by the nuclease.

**Figure 1:**
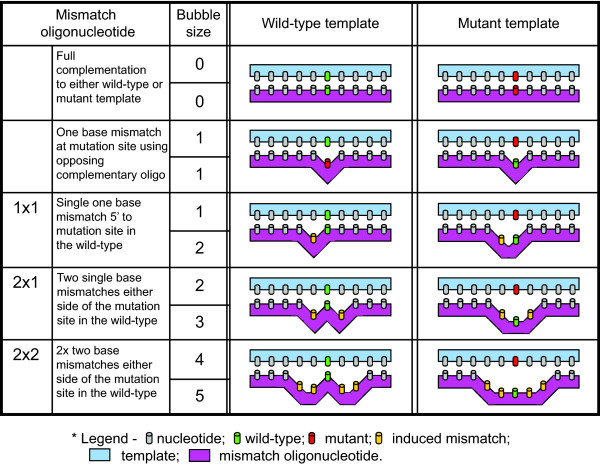
**Schematic representation of mismatch 'bubbles' created by annealing template and mismatch-inducing oligonucleotides**. Mismatches are specifically placed surrounding the mutation site to produce differential binding to the wild-type and mutation templates based solely on the identity of the template. By modifying the position and number of mismatches, different size mismatch 'bubbles' are generated, which may become sensitive to nuclease treatment if sufficient ssDNA is exposed. Here, a stepwise increase in mismatches is explored to determine the minimal 'bubble' size that can confer nuclease sensitivity to the mutant template while maintaining resistance by the wild-type template.

To create oligonucleotides that bind both templates but induce a differential cleavage capacity between wild-type and mutant sequences, mismatches were created in the immediate adjacent nucleotides surrounding the mutation site of the binding oligonucleotide (Figure 1). As the two template sequences (wild-type and mutant) were identical except for the mutation site (A - wild-type; G - mutant), all oligonucleotides were complimentary to the wild-type sequence at the mutation site but mismatched relative to the mutant template (Table 1). For all mismatched oligonucleotide combinations, the mismatch bubbles are larger by one base pair when the oligonucleotides anneal to the mutant sequence, as compared to wild-type sequence, and hence the mutant sequence is rendered more susceptible to cleavage by S1 nuclease than the wild-type sequence. An oligonucleotide designated '1 × 1' produced a single base mismatch when bound to the wild-type sequence, but produced a two base mismatch when bound to the mutant template (i.e., at the mutation position and one position adjacent to it). Similarly, the oligonucleotide '2 × 1' produced a single base mismatch on either side of the mutation site in the wild-type, but produced a three base mismatch (the mutation site plus the positions either side of it) when bound to the mutant template. Finally, the '2 × 2' oligonucleotide produced a two base mismatch on either side of the mutation site in the wild-type sequence, but produced a five base mismatch (the mutation site plus two base positions either side of it) in the mutant template. This therefore produced a series of mismatch 'bubbles' that spanned 0, 1, 2, 3, 4, and 5-bp which could be individually analysed for their sensitivity to S1 nuclease.

**Table 1:** Wild-type and mutant template sequences, and 'bubble-forming' oligonucleotide sequences used in this study

Name	Oligonucleotide sequence (5' > 3')^a, b^
Wild-type sense template	AGATTAAGAGA**A**CCAACACCTCT
Wild-type antisense template	AGAGGTGTTGG**T**TCTCTTAATCT
Mutant sense template	AGATTAAGAGA**G**CCAACACCTCT
Mutant antisense template	AGAGGTGTTGG**C**TCTCTTAATCT
1 × 1 mismatch - sense complimentary	AGAGGTGTTGT**T**TCTCTTAATCT
1 × 1 mismatch - antisense complimentary	AGATTAAGAGA**A**TCAACACCTCT
2 × 1 mismatch - sense complimentary	AGAGGTGTTGT**T**GCTCTTAATCT
2 × 1 mismatch - antisense complimentary	AGATTAAGAGG**A**TCAACACCTCT
2 × 2 mismatch - sense complimentary	AGAGGTGTTAT**T**GTTCTTAATCT
2 × 2 mismatch - antisense complimentary	AGATTAAGATG**A**TAAACACCTCT

^a ^Wild-type and mutant template sequences differ by a single nucleotide change, indicated in bold. Note in the 1 × 1, 2 × 1 and 2 × 2 sequences, the nucleotide at the mutation site is complementary to that of the wild-type sequence.

^b ^Induced nucleotide mismatches to generate mismatch "bubbles" in 1 × 1, 2 × 1, and 2 × 2 oligonucleotides that differ from the template sequence are indicated by being underlined.

### Differential cleavage capacity produced by induced mismatches demonstrates selective degradation of the mutant template

**Figure 2:**
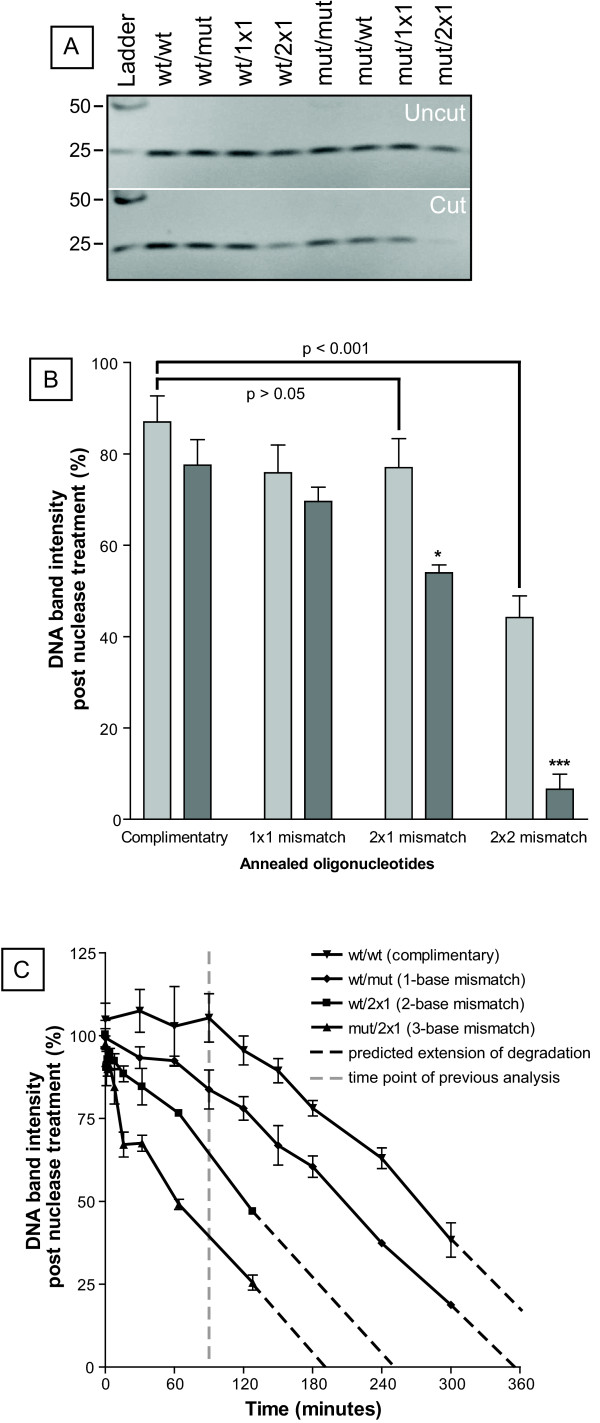
**S1 nuclease treatment of wild-type and mutant templates annealed to mismatch-forming oligonucleotides**. Oligonucleotides annealed at 20°C were incubated with 0.1 U/μl S1 nuclease at 20°C for 90 minutes. (A) Typical polyacrylamide gel electrophoresis depiction of uncut and nuclease-treated annealed oligonucleotides. Ladder: Low Molecular Weight DNA Ladder (NEB) (B) Comparative analysis of nuclease sensitivity against mismatch-inducing oligonucleotides annealed to wild-type (light grey) and mutant (dark grey) templates. One-way ANOVA was used to assess significance of degradation of the wild-type templates as compared to complimentary sequences. Two-way ANOVA was used to assess discrimination between wild-type and mutant templates for each mismatch set (* = p < 0.05; *** = p < 0.001). (C) S1 nuclease cleavage kinetics on stepwise increasing mismatch 'bubble' templates. Annealed oligonucleotides were incubated with 0.1 U/μl S1 nuclease, and incubated at 20°C for the indicated time. Reactions were stopped by adding 1 μl of 0.5 M EDTA and kept on ice prior to electrophoresis. Data is represented as mean percentage of DNA band intensity compared to uncut template ± SEM (N = 3).

An initial S1 nuclease cleavage assay to determine the effects of 'bubble' size on cleavage sensitivity demonstrated that differential cleavage between the wild-type and mutant templates was observed for some, but not all, annealed oligonucleotides (Figure 2A). Both self- and 1 × 1 complementary oligonucleotides showed no significant difference between wild-type and mutant sequences, and both the 1-bp (wild-type) and 2-bp mismatch (mutant) generated by the 1 × 1 oligonucleotide remained resistant to the nuclease (Figure 2B). However, the 2 × 1 oligonucleotide induced a significant difference in cleavage between wild-type and mutant templates (p < 0.05), while maintaining no significant difference between the wild-type/2 × 1 and the wild-type complementary bound templates (p > 0.05). The 2 × 2 bound oligonucleotide produced a greater difference in nuclease sensitivity between the wild-type and mutant template sequences (p < 0.001) and almost complete degradation of mutant template was achieved, however, the wild-type/2 × 2 was also sensitive to the nuclease (approximately 50% reduction relative to the control wild-type complementary template; p < 0.001). Considering one of the primary aims was to minimise wild-type sequence degradation, and that wild-type/2 × 2 was sensitive to nuclease digestion, the 2 × 2 oligonucleotide was discontinued throughout the rest of the study. Theoretically, the wild-type/2 × 2 complex should consist of two mismatched base pairs followed by a matched base pair and then two more mismatches. Given that the dissociation and annealing of dsDNA is a cooperative process, it is likely that the annealed middle position of the wild-type/2 × 2 complex is destabilised by its mismatched neighbours and is, therefore, as susceptible to S1 nuclease digestion as the mutant/2 × 2 complex.

Considering that the 2 × 1 oligonucleotide showed a promising degree of selectivity between wild-type and mutant sequences, it was analysed further over 5-hours in the presence of S1 nuclease (Figure 2C). Wild-type/wild-type, and wild-type/mutant templates were also included in this assay, so that stepwise 0 (wild-type/wild-type), 1 (wild-type/mutant), 2 (wild-type/2 × 1) and 3 (mutant/2 × 1) base mismatches could be analysed simultaneously. Although an increase in 'bubble' size resulted in a stepwise increase in nuclease sensitivity at each time point, the rate of degradation over time was virtually identical for each 'bubble' size, suggesting that the bubble size difference was not influencing the degradation rate as was initially predicted. Furthermore, when the incubation time was extended, both wild-type and mutant sequences were equally sensitive to nuclease, and hence the sequence specificity of the nuclease degradation was lost relative to the 90-minute incubation (Figure 2A, B, C - grey dashed line).

### Novel melting curve assay facilitates determination of Tm and optimal annealing conditions in the presence of increasing mismatched bases

A number of factors, such as DNA concentration, salt concentration, and sequence composition will affect the melting and reassociation kinetics of double-stranded DNA. These characteristics have been used to develop a range of algorithms to estimate the melting temperature (Tm) of perfectly complimentary oligonucleotides (such as those used for PCR primer design); however, there are few algorithms that describe the change of Tm generated by the presence of a single mismatch, let alone multiple mismatches. Furthermore, these algorithms often predict quite different Tm's for a single oligonucleotide pair and thus a calculated Tm is at best an estimate of unknown accuracy. Therefore, a plausible hypothesis for the S1 nuclease degradation of perfectly matched oligonucleotides (Figure 2C) may be imperfect annealing due to uncertainty concerning the real Tm.

**Figure 3:**
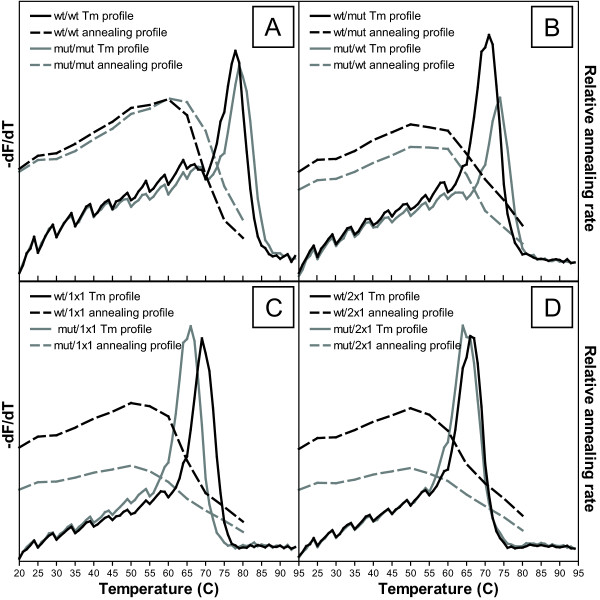
**Fluorometric melting curve analysis of complimentary and mismatched templates to determine optimal Tm and annealing rate**. (A) Full complimentary; (B) reverse single mismatch; (C) 1 × 1 oligonucleotides; (D) 2 × 1 oligonucleotides. Average negative first derivative calculations of normalised fluorescence data was plotted to visualise melting temperature (solid line). Average annealing rate was calculated by measuring the gradient of the logarithmic transformed curve of the normalised fluorescence data across the calculated Tm ± 1°C, and plotted across 5°C intervals spanning 20-80°C (dashed line). Data for both Tm and annealing plots is represented as the mean of three replicates from a single experiment performed, which was typical of three repeated experiments.

To explore this hypothesis, a novel fluorescence-based high resolution melting curve protocol was developed to optimise the annealing conditions empirically by careful measurement of observed Tm's over a range of conditions for the mismatched oligonucleotide sets. This protocol, modified from a conventional melting curve assay, therefore allowed us to determine the optimal annealing temperature (Ta) for each oligonucleotide set. The dsDNA-specific dye, LC Green PLUS, which has been optimised for high resolution melting curve analysis, was used to monitor the disassociation of annealed complexes as the dsDNA is melted into ssDNA. The melting temperature (Tm) of each oligonucleotide mismatch set was determined by calculating the average negative first derivative (-dF/dT) across the annealing temperature range. -dF/dT describes the rate of change of the logarithmic normalised fluorescence data from which the Tm is interpreted as the point at which -dF/dT is at its maximum (Figure 3; -dF/dT). A single base pair increase in mismatch 'bubble' size resulted in an approximate 5°C decrease in Tm (5.0 ± 2.28 SD in observed Tm change) which was similar to the predicted Tm change (5.33 ± 1.09 SD), of which variation in Tm would be due to differences in mismatch base composition and the relative position of the mismatch to the SNP [12]. Additionally, these values for Tm were on average 10.3°C higher than predicted (Table 2 - Ave. ΔTm), which is consistent with an increase in dsDNA template stability in the presence of an intercalating fluorescent dye [13].

**Table 2:** Melting temperature (Tm°C) and annealing temperature (Ta°C) data obtained from fluorometric melting curve analysis

Mismatch Set	Observed Tm	Predicted Tm^a^	ΔTm (Obs -Pred)	ΔTm -ΔTm(Ave)^b^	Optimal Ta	ΔTa (Obs Tm - Ta)	Corrected Ta
wt/wt	78	67	11	0.7	60	18	49.7
wt/mut	71	61.8	9.2	-1.1	50	21	39.7
wt/1 × 1	69	60.2	8.8	-1.5	50	19	39.7
wt/2 × 1	66	56.1	9.9	-0.4	50	16	39.7
mut/mut	79	68.7	10.3	0	60	19	49.7
mut/wt	74	63.1	10.9	0.6	50	24	39.7
mut/1 × 1	66	56	10	-0.3	50	16	39.7
mut/2 × 1	64	51.7	12.3	2	50	14	39.7

Average	5.0 ± 2.28 SD**^c^**	5.33 ± 1.09 SD**^c^**	ΔTm(Ave) = 10.3**^d^**			18.38 ± 3.16 SD**^e^**	

^a ^Predicted Tm was determined using an online Tm calculator (http://unicorn.ps.uci.edu/calculations/DNA/DNAthermocalc.html), which initially calculates a Tm value as if the mismatch was not present, and then subsequently corrects this initial value by the addition of a destabilisation factor dependant on the identity of the mismatch being either a dG, dC, or neither a dG/dC [23].

^b ^ΔTm-ΔTm(Ave) is used as a relative measure of observed annealed complex stability relative to predicted values. A value of zero indicates there is no deviation between observed and predicted Tm values. Annealed templates are considered less-stable as the value becomes more negative, whereas a more positive value indicates greater template stability than predicted.

^c ^Values indicate mean temperature change per single base increase/decrease in "bubble" size.

^d ^Average ΔTm describes the temperature difference as a result of the LC Green dye binding to dsDNA. This value is used to correct the annealing temperature.

^e ^Average of difference in observed Tm and observed annealing temperature, indicative of the decrease away from the Tm needed to achieve optimal annealing conditions.

The optimal annealing temperature of each mismatched oligonucleotide combination was determined by measuring the rate of dsDNA disassociation relative to the observed Tm, i.e., the slope of the gradient formed between Tm ± 1°C in the disassociation profile, across a broad temperature range of 20°C to 80°C in 5°C increments (Figure 3; relative annealing rate). The annealing rate was determined to be almost the same for both wild-type and mutant complimentary template sequences and demonstrated a decrease in annealing rate as the mismatch 'bubble' size increased. The annealing rate was also decreased for the mutant template when compared with the wild-type for both 1 × 1 and 2 × 1 oligonucleotides, indicative of the lower stability of the mutant bound sequence. Therefore, it is clear that the number and type of mismatch affects the reassociation kinetics of oligonucleotide binding and that an increase in base-pair mismatch reduces its ability to hybridise. However, although each mismatch bubble size demonstrated different annealing rates, each oligonucleotide set produced a similar annealing curve, such that an optimal peak height for a given oligonucleotide set was observed at approximately Tm minus 18.38°C ± 3.16 SD (Table 2, Mean ΔTa). Above this temperature, the annealing rate demonstrated a sharp decrease as the annealing temperature approached the Tm, whereas, below this temperature, a comparatively modest decrease in annealing was observed. Therefore, a corrected Ta could be calculated for each oligonucleotide annealing set, which was determined by subtracting the dye-induced temperature increase from the optimal annealing temperature (Table 2, Corrected Ta = [Optimal Ta - AVE ΔTa(10.3)]).

The relative steepness of the gradient used to calculate the optimised annealing temperature is indicative of the thermal stability of the annealed template [14]: as demonstrated in Figure 3, there is an apparent 'trade-off' as the annealing temperature moves away from the optimal Tm. A steeper gradient implies greater stability due to more accurate hybridisation, whereas a shallower gradient implies less stable binding, whether it be due to the incorporation of 'forced' mismatches due to fast annealing as the temperature decreases away from the Tm, or, due to less annealed dsDNA product formed during slower annealing conditions as the temperature increases above the Tm. The data presented were generated using a broad range of temperatures with increments of 5°C. The level of resolution achieved was suitable for the purpose of this investigation and permitted a relatively simple one-step determination of Tm. However, a more finely resolved estimation of Tm and annealing parameters might be necessary to discriminate between more subtle nucleotide changes, such as A/T class IV SNPs, which could be achieved by decreasing the temperature interval and thus permitting the use of smaller temperature increments between data points.

### Optimised annealing conditions enhances stability of nuclease resistant templates and enables efficient nuclease degradation of targeted mutant sequences

**Figure 4:**
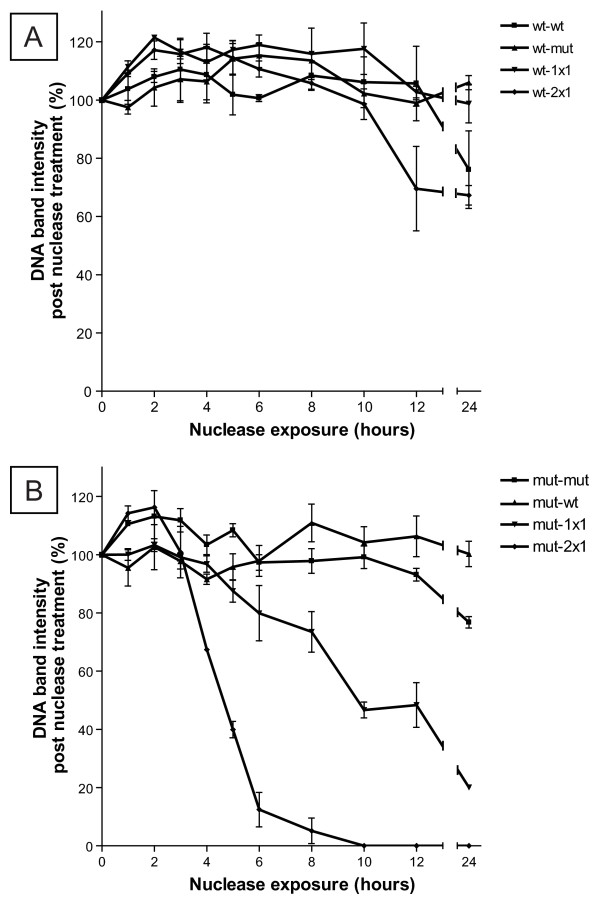
**S1 nuclease cleavage kinetics on stepwise increasing mismatch 'bubble' templates using optimised annealing conditions**. Wild-type (A) and mutant (B) oligonucleotide sets were annealed under specific annealing temperatures determined from the melting curve analysis. Annealed oligonucleotides were incubated with 0.1 U/μl S1 nuclease, and incubated at 20°C for the indicated time. Reactions were stopped by adding 1 μl of 0.5 M EDTA and kept on ice prior to electrophoresis. Data is represented as mean percentage of DNA band intensity compared to uncut template ± SEM (N = 3).

Utilising the empirically determined Ta from the fluorescence assay and data presented in Figure 3 and Table 2, each oligonucleotide combination was annealed and subjected again to nuclease treatment for up to 24 hours (Figure 4). No significant degradation of complimentary and single base mismatch (including reverse complimentary and wild-type/1 × 1) sets for wild-type and mutant oligonucleotides was observed over 24 hours. Some minimal digestion of wild-type/2 × 1 mismatch was observed at 12 and 24 hours. Strikingly however, both mutant/1 × 1 and mutant/2 × 1 annealed templates were significantly digested over the time course in a stepwise manner: the 1 × 1 oligonucleotide was approximately 80% digested after 24 hours by S1 nuclease, whereas the 2 × 1 annealed template was approximately 90% digested after 6 hours and was not detectable after 8 hours. Note that under these conditions, the perfectly annealed (wild-type/wild-type and mutant/mutant) combinations were not detectably digested. A single-base mismatch that was present in both the mixed complimentary and wild-type/1 × 1 complexes was not recognised by S1 nuclease under these conditions, but two consecutive base mismatches were recognised as a ssDNA substrate by S1 nuclease as seen in the mutant/1 × 1 and mutant/2 × 1 templates. Note that two non-consecutive mismatches, as in the wild-type/2 × 1 complex, were not sufficient for S1 nuclease cleavage. The requirement for at least two consecutive mismatches for S1 nuclease cleavage contrasts with several reports of successful cleavage with a single mismatch [2,3,5,15]. However, the observation here may be attributed to the significantly lower enzyme concentration used in this study (0.1 U/μl compared to 2.5-40X higher concentrations reported previously [2,3,5,15]), together with the careful matching of annealing temperature to each oligonucleotide combination allowing for a more stable duplex even in the presence of mismatches.

The dramatic improvement in both the stability of the perfectly annealed templates over a significantly greater time frame (24 hours in Figure 4 compared to the initial time course assay of 6 hours in Figure 2C) and the stepwise increase in degradation rate across the mutant/1 × 1 and mutant/2 × 1 template oligonucleotide combinations highlight the significant effect that the number and type of mismatches have on duplex stability, and emphasise the importance of empirical optimisation of the annealing conditions in hybridisation experiments. Furthermore, these data demonstrate the effectiveness of the novel fluorometric melting curve assay described here.

## Conclusions

The work presented in this investigation demonstrates a new set of conditions under which S1 nuclease can be rendered sequence specific (i.e., the creation of an 'artificial restriction enzyme' of predetermined sequence specificity). As well as meeting the requirement for discrimination of a single base point mutation, the method described provides a simple mechanism to vary the rate of cleavage dependent on the orientation of the mismatch relative to the mutation site and requires only transient binding of the oligonucleotide to the template while the S1 nuclease is present to cleave the substrates. The generation of S1 nuclease sensitive mismatch 'bubbles' is likely to have a broader application than mtDNA haplotype discrimination alone, including alternative SNP detection strategies and other manipulations that require sequence specific cleavage of DNA, such as the isolation of specific genomic sequences for cloning in the absence of unique restriction sites. The fluorometric assay presented was able to both rapidly and simultaneously determine Tm and annealing conditions for complimentary and mismatched oligonucleotides. Of broader interest, this new fluorescence assay can be used independently of the S1 nuclease assay, providing an efficient hybridisation analysis protocol (i.e., 96-well plate format as demonstrated here) that may supersede traditional assays, such as filter-hybridisation- [16,17] and sequencing-based radiolabelling [18], and temperature-controlled spectrophotometry [19-21]; all of which are labour intensive and require specialised equipment. Furthermore, this methodology has the potential to be routinely used to determine optimum Tm and annealing conditions for complimentary and mismatched oligonucleotides in a range of applications that rely on accurate hybridisation, such as oligonucleotide- (microarrays) and probe-based assays (real-time PCR, fluorescence *in situ *hybridisation), or, protocols that assess the level of degenerate hybridisation, such as SNP detection/screening or evolutionary divergence in comparative taxonomical studies.

## Methods

### Materials

Cartridge-purified oligonucleotides were obtained from Invitrogen and dissolved in sterile water. Oligonucleotide concentration was determined using a NanoDrop 1000 spectrophotometer and was subsequently adjusted to a working concentration of 100 ng/μl and stored at -20°C. S1 nuclease was obtained from Promega. LC Green PLUS was obtained from Idaho Technologies Inc.

### Complementary oligonucleotide design

Oligonucleotides were designed to complement both sense and antisense strands extending 11-bp either side of the A8344G mitochondrial MERRF mutation site to create a 23-bp complimentary oligonucleotide (See Table 1 for oligonucleotide sequences used). The '1 × 1' oligonucleotides were identical to the wild-type sequence but contained a 5' G > T (sense complimentary) and 3' C > T (antisense complimentary), creating a single 1-bp mismatch in the wild-type and a 2-bp mismatch in the mutant template. The '2 × 1' oligonucleotides were homologous to the wild-type sequence but contained a 5' G > T and 3' T > G (sense complimentary) and 5' A > G and 3' C > T (antisense complimentary), creating a 1-bp mismatch either side of the adenosine in the wild-type sequence and a 3-bp mismatch in the mutant template. The '2 × 2' oligonucleotides were homologous to the wild-type sequence but contained a 5' GG > AT and 3' TC > GT (sense complimentary) and 5' GA > TG and 3' CC > TA (antisense complimentary), creating a 2-bp mismatch on both sides of the adenosine in the wild-type and a 5-bp mismatch in the mutant template. The difference in induced mismatches observed between mutant and wild-type sequence is schematically represented in Figure 1.

### Oligonucleotide binding and cleavage assays

Complementary oligonucleotides were used to model the formation of nuclease sensitive ssDNA 'bubbles'. As each combination of 1 × 1, 2 × 1 and 2 × 2 oligonucleotides could be annealed to both sense and antisense strands of both wild-type and the mutant sequence, it was possible to generate and subsequently analyse a simulation of complimentary binding of the 'bubble' inducing antisense oligonucleotides to both strands within a double stranded DNA template. The following protocol was used unless otherwise stated: 200 ng of each oligonucleotide (sense and antisense) were mixed with hybridisation buffer (final concentration; 800 mM NaCl, 10 mM Tris-HCl, 1 mM EDTA, pH 8.0 [22]) in a total of 10 μl in microcentrifuge tubes. Samples were placed in a 95°C heat block for 5 minutes and then rapidly cooled to the designated annealing temperature in a heat block for 120 minutes to anneal complementary oligonucleotides. Annealed oligonucleotides were kept on ice until further use. Post-annealing, each 10 μl sample subsequently received 1 μl of 10X S1 nuclease buffer and either 1 μl of S1 nuclease (treated samples; 0.1 U/μl working concentration), or, 1 μl H_2_O (untreated). All samples were incubated at 20°C for the defined time-period, after which the cleavage reaction was terminated by the addition of 1 μl EDTA (0.5 M stock) and placed at -20°C until further analysis. To each sample, 2.5 μl of 6X loading dye was added, after which the entire volume was subsequently loaded onto a 16% (w/v) 19:1 (acrylamide:bis) Tris/Borate/EDTA (TBE) polyacrylamide gel and run for 150 minutes at 100V. Gels were washed, stained in ethidium bromide, and viewed by UV illumination. Images of gels were further analysed using ImageJ (http://rsbweb.nih.gov/ij/), whereby relative DNA band intensities were semi-quantitatively assessed by measuring the pixel intensity of nuclease-treated samples compared against untreated controls.

### Oligonucleotide Tm and annealing analysis

Oligonucleotide binding conditions were optimised by fluorometric assessment of melting curves performed on the Roche LightCycler 480 Real-Time PCR System configured for melting curve analysis (Software release 1.5.0) using the dsDNA intercalator LC Green PLUS. For each oligonucleotide annealing reaction, 200 ng of each sense and antisense oligonucleotide were combined with 2 μl 5X hybridisation buffer (see above), 1 μl of 10X LC Green, and made up to a final volume of 10 μl with H_2_O. Samples were loaded onto a 96-well plate in triplicate, and were kept on ice and protected from light until being loaded onto the LightCycler 480. Run cycling parameters are outlined in Additional file 2. In brief, samples were initially heated to 95°C for 1 second to initiate dsDNA strand disassociation, which was followed by cooling to 20°C to initiate annealing. Samples were held at 20°C for 10 minutes to facilitate annealing of the oligonucleotides, before heating back to 95°C. A single fluorometric data point was collected for every 1°C increment as the temperature was raised back to 95°C. The temperature was then decreased to 25°C, after which the cycle was repeated, increasing the annealing temperature by 5°C after each cycle, until a final annealing temperature of 80°C was reached. The samples were cooled to a final resting temperature of 35°C (see Additional file 3 for a schematic diagram of cycling conditions).

### Fluorometric data analysis

Raw high-resolution fluorometric melting data were exported from the LightCycler 480 into Microsoft Excel for further analysis. The data for each oligonucleotide set were collated and arranged as annealing temperature along the X-axis (rows; 5°C increments from 20°C to 80°C) and the fluorescence data collected as each single data point per °C along the Y-axis (columns; single degree increments from 20°C to 95°C), after which a mean fluorescent value was calculated from the combined triplicate samples. To determine the melting temperature (Tm) value for each annealed oligonucleotide set, the mean fluorescence intensity values were first normalised, so that within each sample, a normalised fluorescent data point represented a percentage of the highest fluorescent value collected for the particular annealed oligonucleotide set. The negative first derivative of each fluorescence acquisition point was generated using the following Excel equation:

$$-\text{dF/dT}=\text{SUM}\left(\left(\left(\text{LOG}\left({\text{X}}_{\text{n}}\right)\right)-\left(\text{LOG}\left({\text{X}}_{\text{n}-\text{1}}\right)\right)\right)*-\text{1}\right)$$

where X_n _is equal to a particular y-axis fluorescence value, and X_n-1 _is equal to the fluorescence value preceding the X_n _y-axis value. The mean of all negative first derivatives for each annealing temperature (across the x-axis) was plotted to determine the Tm.

An annealing efficiency profile for each annealed oligonucleotide mismatch was calculated to determine the optimal annealing temperature required for precise annealing in the presence of base pair mismatches. The profile for each oligonucleotide set was determined by converting the normalised fluorescent data described above into logarithmic values, and the gradient of the slope across the experimentally determined Tm ± 1°C for each annealing temperature was calculated using the following Excel equation:

$$\text{Slope}=\text{SUM}(\left(\left(\text{LOG}\left({\text{X}}_{\text{Tm}-\text{1}{}^{\circ}\text{C}}\right)\right)-\left(\text{LOG}\left({\text{X}}_{\text{Tm}+\text{1}{}^{\circ}\text{C}}\right)\right)/\text{3}\right)$$

where X_Tm-1°C _represents the Tm minus 1°C and X_Tm+1°C _represents the Tm plus 1°C. From the profile generated, the most efficient annealing conditions and hence the optimal annealing temperature (Ta) was determined by the peak maximum.

### Data and statistical analysis

Graph construction and their associated analyses (one-way ANOVA, two-way ANOVA) were performed using GraphPad Prism (version 5.0). Manipulation of fluorescent melting curve data, including calculations of melting and annealing profiles and their associated analyses, were performed in Microsoft Excel (2003).

## Abbreviations

SNP: single nucleotide polymorphism; dsDNA: double-stranded DNA; ssDNA: single-stranded DNA; RFLP: restriction fragment length polymorphism; PNA: peptide nucleic acid; Tm: melting temperature; -dF/dT: negative first derivative; MERRF: Myoclonic epilepsy and ragged-red fibre; Ta: annealing temperature

## Authors' contributions

SRD conceived the study, carried out the experiments and analysed the data, and drafted the manuscript. CKC and WNG helped refine the project idea and study design, and in drafting the final manuscript. All authors read and approved the final manuscript.

## Supplementary Material

Additional file 1 **Supplementary Figure 1: *Trans- *versus *cis-*orientated binding of molecules to generate regions of ssDNA**. Figure demonstrates two different approaches to generate mismatch bubbles based on the orientation of the binding oligonucleotides, which can act as substrates for an ssDNA endonuclease. Click here for file

Additional file 2 **Supplementary Table 1: Cycling conditions used to generate melting curve profiles**. Table contains the specific temperature and data acquisition conditions for generating melting curve profiles. Click here for file

Additional file 3 **Supplementary Figure 2: Schematic representation of cycling conditions used to generate melting curve profiles**. Figure provides a schematic representation of the of temperature changes and data acquisition during melting curve analysis. Click here for file
