# Supplementary material for: Enhanced annealing of mismatched oligonucleotides using a novel melting curve assay allows efficient in vitro discrimination and restriction of a single nucleotide polymorphism
Source: BMC Biotechnol. 2011 Aug 30;11:83. doi: 10.1186/1472-6750-11-83 (PMC3175457; doi:10.1186/1472-6750-11-83)
Supplement: Additional file 2 — Supplementary Table 1: Cycling conditions used to generate melting curve profiles. Table contains the specific temperature and data acquisition conditions for generating melting curve profiles. [file 1472-6750-11-83-S2.DOC]

**Supplementary Table 2.** Temperature and data acquisition conditions for generating melt curve profiles.

| **Target (°C)** | **Acquisition Mode** | **Hold**  **(hh:mm:ss)** | **Temperature ramp rate (°C/s)** | **Data acquisitions**  **(per °C)** |
| --- | --- | --- | --- | --- |
| 95 | None | 0:00:01 | 4.4 | - |
| 20 | None | 0:10:00 | 2.2 | - |
| 95 | Continuous | 0:00:01 | 0.57 | 1 |
| 25 | None | 0:10:00 | 2.2 | - |
| 95 | Continuous | 0:00:01 | 0.57 | 1 |
| 30 | None | 0:10:00 | 2.2 | - |
| 95 | Continuous | 0:00:01 | 0.57 | 1 |
| 35 | None | 0:10:00 | 2.2 | - |
| 95 | Continuous | 0:00:01 | 0.57 | 1 |
| 40 | None | 0:10:00 | 2.2 | - |
| 95 | Continuous | 0:00:01 | 0.57 | 1 |
| 45 | None | 0:10:00 | 2.2 | - |
| 95 | Continuous | 0:00:01 | 0.57 | 1 |
| 50 | None | 0:10:00 | 2.2 | - |
| 95 | Continuous | 0:00:01 | 0.57 | 1 |
| 55 | None | 0:10:00 | 2.2 | - |
| 95 | Continuous | 0:00:01 | 0.57 | 1 |
| 60 | None | 0:10:00 | 2.2 | - |
| 95 | Continuous | 0:00:01 | 0.57 | 1 |
| 65 | None | 0:10:00 | 2.2 | - |
| 95 | Continuous | 0:00:01 | 0.57 | 1 |
| 70 | None | 0:10:00 | 2.2 | - |
| 95 | Continuous | 0:00:01 | 0.57 | 1 |
| 75 | None | 0:10:00 | 2.2 | - |
| 95 | Continuous | 0:00:01 | 0.57 | 1 |
| 80 | None | 0:10:00 | 2.2 | - |
| 95 | Continuous | 0:00:01 | 0.57 | 1 |
| 35 | None | 0:00:01 | 2.2 | - |
